# Reconstruction of Segmental Duplication Rates and Associated Genomic Features by Network Analysis

**DOI:** 10.1093/gbe/evaf011

**Published:** 2025-02-21

**Authors:** Eldar T Abdullaev, Dinesh A Haridoss, Peter F Arndt

**Affiliations:** Department of Computational Molecular Biology, Max Planck Institute for Molecular Genetics, Berlin, Germany; Department of Computational Molecular Biology, Max Planck Institute for Molecular Genetics, Berlin, Germany; Department of Electrical Engineering and Computer Science, Indian Institute of Science Education and Research Bhopal, Bhopal, India; Department of Computational Molecular Biology, Max Planck Institute for Molecular Genetics, Berlin, Germany

**Keywords:** segmental duplications, complex networks, high-copy repeats

## Abstract

Segmental duplications are long genomic duplications that are fixed in a genome. Segmental duplications play an important evolutionary role because entire genes can be duplicated along with regulatory sequences. The ancestral segmental duplications of the human lineage gave rise to genes that are involved in the development of the human brain and provided sites for further genomic rearrangements. While some duplicated loci have been extensively studied, the universal principles and biological factors underlying the spread of segmental duplications remain unclear. Here, we represent segmental duplications in a network, with edges corresponding to duplication events and nodes corresponding to affected genomic sites. This representation allowed us to estimate how many duplications had occurred at each locus, and thereby enabling the prediction of genomic features associated with increased duplication rates. Our comprehensive study of genomic features associated with duplications and those associated with increased duplication rates allowed us to identify several biological factors affecting a segmental duplication process. In our study, we describe genomic features associated with increased duplication rates, three signatures of the duplication process and associations of segmental duplications with different classes of high-copy repeats. Furthermore our method is readily implemented and can easily be applied to segmental duplications of other genomes to build a network of segmental duplications or to predict real duplication events.

**Significance**

Segmental duplications play an important role in genome evolution and disease development. However, there is little known about the genomic drivers of duplication events. We performed a comprehensive analysis of the genomic features that influence the duplication process using complex network approach. We generated a catalog of sequence features and sequence repeats that increase duplication rates. Additionally, we delineated distinctive patterns of duplication events observed in the human genome, which we termed “duplication signatures.”

## Background

Segmental duplications (SDs) or low-copy number repeats are long duplications of genomic sequence that are fixed in a population. Conventionally, only those duplications longer than 1 kbp and with the level of sequence identity >90% are annotated as segmental duplications. Assuming a clock-like mutation rate in the human genome, the SDs defined this way correspond to duplication events that happened after the divergence of the New and Old World monkeys (Bailey and Eichler 2006; She et al. 2006) about 40 Myr ago. Based on the relative position of the two copies of SDs they are classified as intrachromosomal (when both copies are located on the same chromosome) or interchromosomal (when those are on different).

SDs are very unevenly distributed in the human genome. Some loci are extensively duplicated while others are depleted of segmental duplications. For example, pericentromeric and subtelomeric parts of chromosomes are enriched with SDs, especially with interchromosomal ones (Guy et al. 2003; She et al. 2004; Linardopoulou et al. 2005). One effort to study the global principles of the SDs evolution was made by Jiang et al. (2007). Complex duplicated regions were divided into duplication subunits (regions of continuous synteny) and their evolutionary history was analyzed independently. This allowed the observation of so-called “core duplicons”: sequences that are focal points of multiple duplications in the human genome. Core duplicons are often associated with gene families under strong positive selection. Alternatively, the general principles of the segmental duplication process were studied using the complex network approach (Abdullaev et al. 2021). A network of segmental duplications where nodes represent genomic loci involved in duplications, while edges correspond to alignments between loci was constructed and it’s growth was modeled. As a result, a model where duplication rates preferentially grow with node degrees (the preferential copying model or PCM) is the one according to which segmental duplications were accumulated in the human genome and probably in the genomes of other vertebrate species (Abdullaev et al. 2021). Apart from these rather universal dynamic principles of SDs evolution, there are many genomic features that influence duplication rates.

A slight positive correlation of SDs with the gene density along with negative correlation with recombination rates was reported by Zhang et al. (2005) soon after the completion of the Human Genome Project and comprehensive SDs annotation. Moreover, large duplications tend to be enriched in heterochromatic parts of the genome or, according to other observations, in hetero- to euchromatin transition regions (Grunau et al. 2006; Kirsch et al. 2008). Duplicated regions have on average a higher G/C content compared to the rest of the genome (Bailey et al. 2003; Zhang et al. 2005). Consistent with this, breakpoints of copy number variations (CNVs) appear to be enriched in G/C-rich sequences, which, according to some predictions, may form G-quadruplexes (Bose et al. 2014). These factors were observed on an irregular basis and not necessarily reproduced in other experimental settings. The Telomere-to-Telomere consortium has also identified other characteristic features of segmental duplications in their efforts to assemble complete human genomes from the telomere to the telomere (Nurk et al. 2022). For example, increased single nucleotide variants (SNVs) density, higher G/C and CpG content than in nonduplicated sequences (Harpak et al. 2017; Vollger et al. 2023). Partly these features were attributed to extensive nonallelic gene conversion. The recent estimate reports that 34% of SD sequence was affected by nonallelic gene conversion in at least one of the 102 haplotypes analyzed (Vollger et al. 2023).

High-copy repeats are often causal for segmental duplications and therefore are often enriched at SD breakpoints. Some cases of duplication causing repeats were reported even before a genome-scale SDs annotation in the human genome (Eichler et al. 1996, 1999; Guy et al. 2000). The systematic study by Bailey et al. (2003) identified those repeat classes that are significantly overrepresented at SDs breakpoints when compared with the genome average. Only accurately annotated SDs that did not overlap with other duplications were analyzed. Two repeat classes are significantly overrepresented at breakpoints: *Alu* repeats and satellites (specifically, *HSATII*, *GSAT*, and *TAR1*), while many repeat classes are even underrepresented in flanking regions. Specifically, among *Alu* subfamilies, younger ones (*AluY* and *AluS*) account for the enrichment, while the oldest primate subfamily (*AluJ*) does not (Bailey et al. 2003). The burst of *Alu* retroposition around 35 to 40 Myr made an ancestral human genome more susceptible to segmental duplications (Bailey et al. 2003; Bailey and Eichler 2006). Later resulting SDs themselves became a source of homology for further duplication events.

Replication timing is another factor associated with duplications. For example, it was observed that recurrent CNVs are enriched in early replicating genomic sites, while nonrecurrent ones are in late replicating regions (Koren et al. 2012; Chen et al. 2015). Other than that, some groups observed enrichment of CNVs in early replicating regions (Lu et al. 2014), while other in late ones (De and Michor 2011). Finally, it was suggested that division into early and late replicating regions is not sufficient to explain CNVs distribution. The hotspots of CNVs are significantly associated with sites of reduced DNA polymerase velocity (those where the difference in replication timing is the largest), in other words, in transition regions located between early and late replicating sites (Chen et al. 2015).

In this study, we are focused on finding genomic features affecting duplication rates. To solve this task, we exploit the network representation of human SDs which we described above. Firstly, based on characteristics of the SD network, we approximately evaluate the number of duplication events that each duplicated region (or node of the SD network) has undergone in the past evolution. Then we analyze associations of genomic features with duplication rates for those regions. Finally, we looked at characteristic patterns or signatures of SDs.

## Results

### The SD Network Construction

**Fig. 1.**
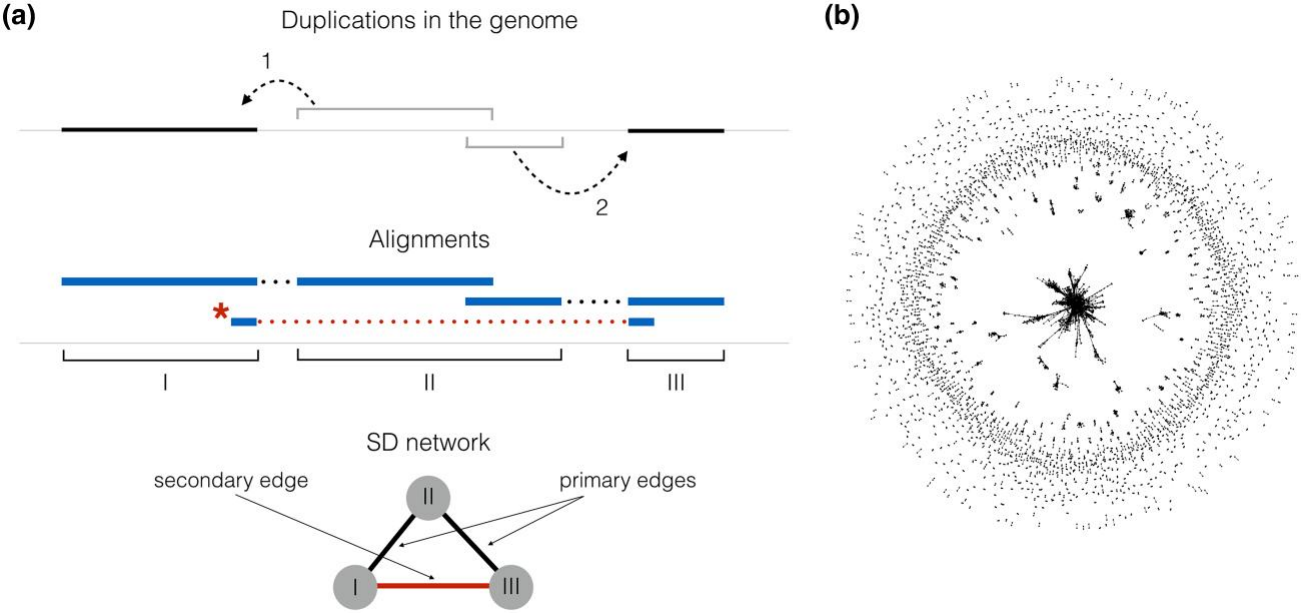
a) A simple example of two duplication events in a genome and an SD network that corresponds to them. Duplication events are marked with Arabic numerals while resulting groups of overlapping alignments (duplicated regions) with roman ones. Our two duplications result in two primary and one secondary (marked with the asterisk) alignments. The last one does not correspond to a duplication event and appears as a result of overlapping duplication events. The SD network is constructed in the following way: nodes represent duplicated regions (marked with respective roman numerals), edges are added if an alignment between two duplicated regions exists. b) The network of segmental duplications. The SD network includes a giant component (in the center) and multiple smaller connected components (Abdullaev et al. 2021).

In our analysis, we used annotated SDs of the human genome (Bailey et al. 2001, 2002). The annotation includes a list of pairwise alignments between regions of the GRCh38 reference genome which are longer than 1 kbp with at least 90% of sequence identity between copies. Not all such alignments correspond to actual duplication events, some of them appear as a result of overlap between an already duplicated region and a new SD (Fig. 1a). As a result a new copy is homologous to both an ancestral region and its another copy. However, only the first of two alignments correspond to an actual duplication event. We call this type of alignments “primary” as opposed to “secondary” alignments which appear because of overlaps between duplications and not correspond to a duplication event between a pair of sequences.

From all human autosomal SDs, we generated the network of segmental duplications or the SD network as described in Abdullaev et al. (2021) (Fig. 1b). Each node of the network represents a duplicated region, which we define as a genomic interval that covers a maximal set of overlapping alignments. If, for example, copies of a tandem duplication overlap, they would be represented as a single node in the network. In other words, a duplicated region equals to a union of all overlapping alignment intervals. Undirected edges are added if alignment between two corresponding duplicated regions (nodes) exists (Fig. 1a). The resulting SD network includes 6,656 nodes and 16,042 edges, which are organized in 1,999 connected components. Among those components one stands out in the size. The giant component contains 1,325 nodes (19.9% of all nodes) and 9,678 edges (60.3% of all edges). All self-loops and multiple edges were filtered out from the SD network.

We analyzed the community structure of the SD network. Communities are densely connected groups of nodes that, from a biological perspective, are likely to have a common evolutionary history. We used the label propagation algorithm described by Raghavan et al. (2007) to predict the communities of nodes in the SD network. In total there were 2,071 communities, most of which corresponded to small connected components. Of all the communities detected, 54 were observed in the giant component. To gain biological insight into the nature of the observed node communities, we overlapped our duplicated regions with the core duplicons from Jiang et al. (2007). For each of the 13 genes overlapping core duplicons (focal points of an active duplication process), we extracted all duplicated regions containing these duplicons and looked at how these regions mapped onto our SD network. Of all the nodes that overlapped core duplicons, the majority (68%) were mapped on the giant component (which itself contains 20% of all nodes). Interestingly, we found that in most cases duplicated regions sharing core duplicons belong to one or two shared communities, while duplicated regions associated with different core duplicons mostly belong to different node communities (supplementary fig. S7, Supplementary Material online). In principle, by analyzing the community structure of the SD network alone, one can make predictions (or reduce the search space) about the core duplicons operating in any genome of interest with well-annotated SDs.

As described above, edges of the SD network represent alignments of homologous sequences that share a common origin; however, the information on the temporary order and direction of duplications (i.e. which genomic region among two copies is ancestral) is missing. Moreover, edges of the SD network represent either real duplication events or “secondary” alignments that appear because of overlaps between independent duplications. Reconstruction of real duplication events from the whole network of duplicated regions would allow us to further look into biological factors responsible for segmental duplications.

### Duplication Event Reconstruction: Graphs Without Cycles

Earlier we found that the PCM explains many features of the SD network topology (Abdullaev et al. 2021). Thus we can formulate some hypotheses about the origin of cycles in the network of segmental duplications. Cycles in PCM simulated networks appear as a result of duplication when a new “daughter” node inherits an edge to a neighbor of the “mother” node from it (supplementary fig. S1a, Supplementary Material online). Since it is the only way how a daughter node inherits edges one expects to see only cycles of length K=3. It is impossible to acquire a neighbor from a mother node that does not have one, that is why we do not get longer cycles (supplementary fig. S1b, Supplementary Material online). We analyzed cycles and shortest self-paths (shortest path from a node to itself where exists) of the SD network. In agreement with this, we observe that our SD network is depleted with cycles of size >3 and shortest self-paths longer than three edges in comparison with other networks of well-known topology (supplementary table S1, Supplementary Material online).

**Fig. 2.**
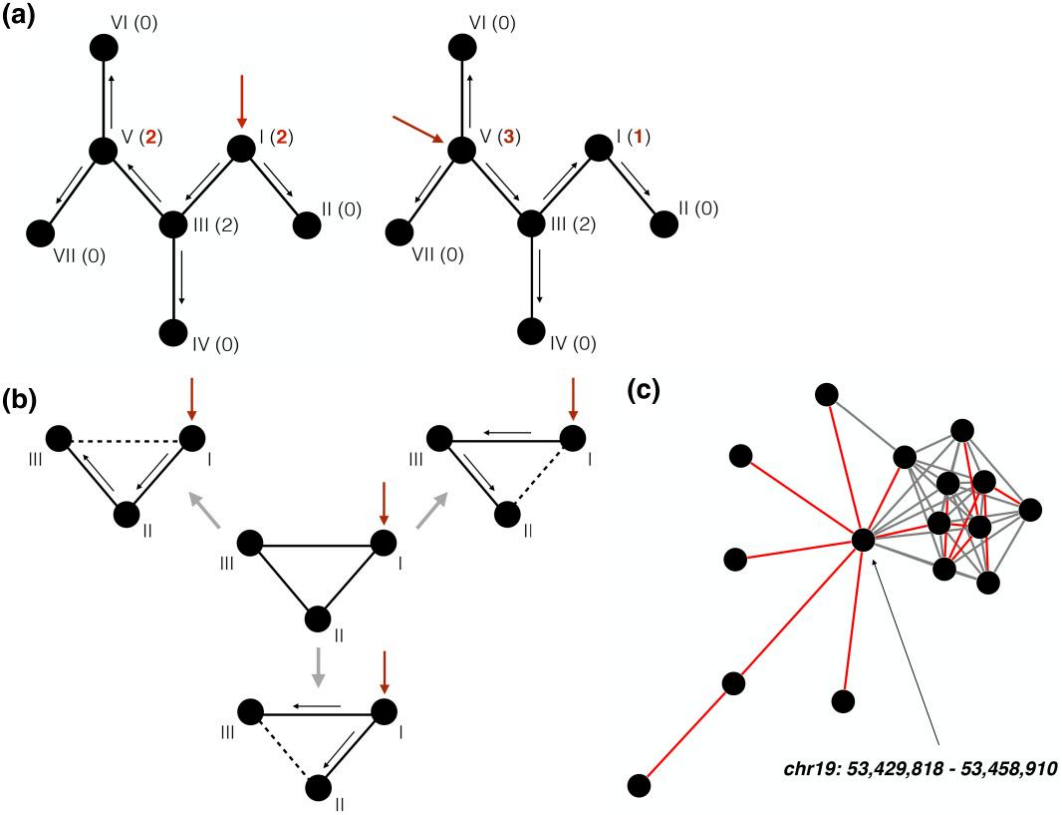
a) Reconstruction of duplication events is a straightforward task when cycles are absent. Numbers in parentheses show number of duplications for each node, black arrows represent duplication events (pointing from “mother” to “daughter” nodes), red arrows point to the starting nodes while the dotted lines represent secondary edges. Assigning another starting node results in alternative duplication history, however, overall number of duplications each node made stays almost intact (differs only for starting nodes). The cyclic graph in the center of the scheme (b) can be reconstructed in three different ways (each of three edges in the cycle can be secondary). The scheme (c) illustrates the reconstruction of duplication events (red edges) in one connected component of the SD network. The subgraph of reconstructed duplications must have a tree structure (be connected and acyclic).

Let us consider a case of duplication events reconstruction from an example duplication network without any cycles. If we disallow any inheritance of neighbors (e.g. by simulating PCM with f=0), we will observe a network without any cycles where each edge represents a real duplication event. Directionality of duplication events (mother to daughter nodes relationship) can be reconstructed in a unique way only if we know which node was the ancestral one for each connected component (Fig. 2a). By comparing two acyclic graph scenarios, we can see that direction of edges changes when taking another ancestral node while the number of duplications that each node has undergone is almost invariant to this choice. Thus for our task it is sufficient to find edges in the SD network that correspond to real duplications while assigning directionality is not needed. In other words, resolving cycles in the SD network by excluding all secondary edges would be enough to say how many times each node duplicated in the course of the network growth.

### Duplication Event Reconstruction: Graphs with Cycles

The only way how cycles of size three can appear in a PCM generated network is when a daughter node inherits an edge from a mother node. This can happen in several scenarios (Fig. 2b) so reconstruction of duplication events from a network with cycles is not a trivial task anymore. It can be formulated as a search for a subgraph tree (a spanning tree) that goes through all nodes of the SD network and covers only those edges that correspond to real duplication events (Fig. 2c). The spanning tree by definition does not have any cycles, thus for the SD network with $N_{\mathrm{SD}}=6,656$ nodes, $E_{\mathrm{SD}}=16,042$ edges, and $C_{\mathrm{SD}}=1,999$ components we expect a spanning tree with $N_{\mathrm{STree}}=N_{\mathrm{SD}}=6,656$ nodes, $E_{\mathrm{STree}}=N_{\mathrm{SD}}-C_{\mathrm{SD}}=4,657$ edges, and $C_{\mathrm{STree}}=C_{\mathrm{SD}}=1,999$ components. There are several existing algorithms that can find the minimum spanning tree (MST) for a graph that goes through edges of minimal overall weight. To apply the algorithms, one has to assign weights to the edges of the SD network, such that lower weights correspond to likely primary edge. We use an heuristic approach to assign such weights to the network of SDs. Our approach is based on a set of assumptions. First of all, we study each node of the SD network independently. Secondly, according to the PCM each node of a connected component except for the ancestral pair of nodes appears as a result of duplication of its mother node. Moreover, a mother node has to be among the neighbors of a node of interest. Finally, it is expected that a daughter node shares the highest fraction of its neighbors with a mother node. With these three assumptions, we can assign edge weights as further detailed in the corresponding section of the Supplementary materials.

In practice, our approach was implemented as the following weighting scheme. For each edge *e* of the SD network connecting nodes *a* and *b*, we assigned the weight $w_{e}=1-\frac{\left\|N(a)\cap \;N(b)\right\|}{\min(k_{a},k_{b})}$, where N(⋅) is a set of neighbor nodes of a node of interest, ‖⋅‖ denotes the number of elements in a set, and *k* is a node degree. Values $w_{e}$ lie in the interval $\left[0<w_{e}\leq 1\right]$ where $w_{e}=1$ when no neighbors are shared, while $w_{i}>0$ because there is always at least one neighbor not included in the overlapping set N(a)∩N(b). In other words, ‖N(a)∩N(b)‖<min(‖N(a)‖,‖N(b)‖) since *a* is not in N(a) and *b* is not in N(b) by definition. As a result, edges connecting nodes with many shared neighbors are of lower weight and thus more likely being included in the MST. These edges are of our interest, because likely correspond to real duplication events.

### Accuracy Evaluation

We tested the accuracy of our approach on PCM simulated networks. To make the PCM model more consistent with the real life events, we added processes corresponding to loss of alignments in a clock-like manner and gene conversion events (see Methods). Two metrics were used to measure the accuracy of our predictions: the fraction of correct (primary) edges in the MST and a variance explained in duplications number that each node has undergone during the simulation (Table 1). We compared our method, listed as “Predicted MST,” with several alternative strategies of predicting number of duplication events per node (listed in the Table 1). These include multiple node centrality measures. We also considered two trivial models: simple use of PCM network node degrees and a random spanning tree. These models define baseline accuracy levels. Our method performs the best and explains 67% of variance in the number of duplications while trivial use of node degrees gets only 52%. Other than that, we correctly identify 62% of primary edges while a random spanning tree, per average, passes through only 13% of primary edges.

**Table 1.** We compared several approaches for predicting the actual number of duplications using PCM simulated networks

Method	Edges match (%)	Variance explained ($R^{2}$)
Predicted MST	62	0.67
Node degrees vector	NA	0.52
Random spanning tree	13	0.33
Betweenness	NA	0.57
Closeness centr.	NA	0.08
Eigenvector centr.	NA	0.41
PageRank centr.	NA	0.55
Radiality centr.	NA	0.06

To evaluate an accuracy, we measured the fraction of correctly predicted primary edges (where possible) and a variance explained for the vector of real duplications number. We measured an accuracy of our MST based algorithm described in the main text (“Predicted MST” row in the table) along with multiple centrality measures (Brin and Page 1998; Seth Bromberger 2017). We also considered the vector of node degrees and a random spanning tree covering nodes of a synthetic network as a trivial models to estimate a baseline quality of predictions.

We used this method to distinguish primary from secondary duplications in the SD network. Using the resulting MST, we measured the number of duplication events that occurred in each duplicated region. To validate the accuracy of our prediction, we further analyzed characteristics of edges predicted as primary duplications. For example, matching coordinates of a pair of SDs is a characteristic feature of a secondary alignment. These “suspicious” alignments often appear as a result of overlap between duplication events (see supplementary fig. S2a, Supplementary Material online). We observed that “suspicious” edges with matching breakpoints were depleted in our predicted MST when comparing with random samples of edges from the rest of the network (empirical *p*-value <0.0001). Secondly, we expect that a correct MST covering real duplication events includes more edges of higher sequence identity than a random set of edges from the SD network. We found that our predicted MST for the SD network is enriched with highly identical edges (alignments). There are more alignments of sequence identity higher than 0.99 in the predicted MST in comparison with random samples of edges from the rest of the SD network (empirical *p*-value =0.01). We explained this effect in more detail in supplementary fig. S2b, Supplementary Material online. Same properties were observed when we filtered out duplicated regions neighboring assembly gaps (supplementary fig. 4a and b, Supplementary Material online). This suggests that the above distributions are not due to biases associated with the assembly incompleteness. When building the resulting MST of primary duplications, we manually modified the weights of “suspicious” edges so that none of them were included in the MST of primary duplication events. Only edges with matching breakpoints bordering assembly gaps were retained, as these matching breakpoints are likely to arise from the incompleteness of the genome assembly.

### Associations with Genomic Features

#### Genomic Features Associated with Duplicated Regions

For each duplicated region (node) of the SD network, we collected multiple genomic features associated with that region. These genomic features include: replication timing, recombination rates, openness of chromatin, genome assembly gaps, CTCF binding sites, coordinates of a duplicated region, G/C nucleotides content, number of gene overlaps, repeat overlaps, and CpG island overlaps (see supplementary table S2, Supplementary Material online for details). Some features were measured inside the duplicated regions (between the breakpoints), while some in flanking regions of length 50 bps (“Position” column at supplementary table 2, Supplementary Material online). We used those features to find associations with the number of duplications that we predicted during the reconstruction step. In other words, we formulated a machine-learning task of predicting the number of duplications (response variable) given the matrix of genomic features associated with corresponding duplicated regions (predictor variables).

**Fig. 3.**
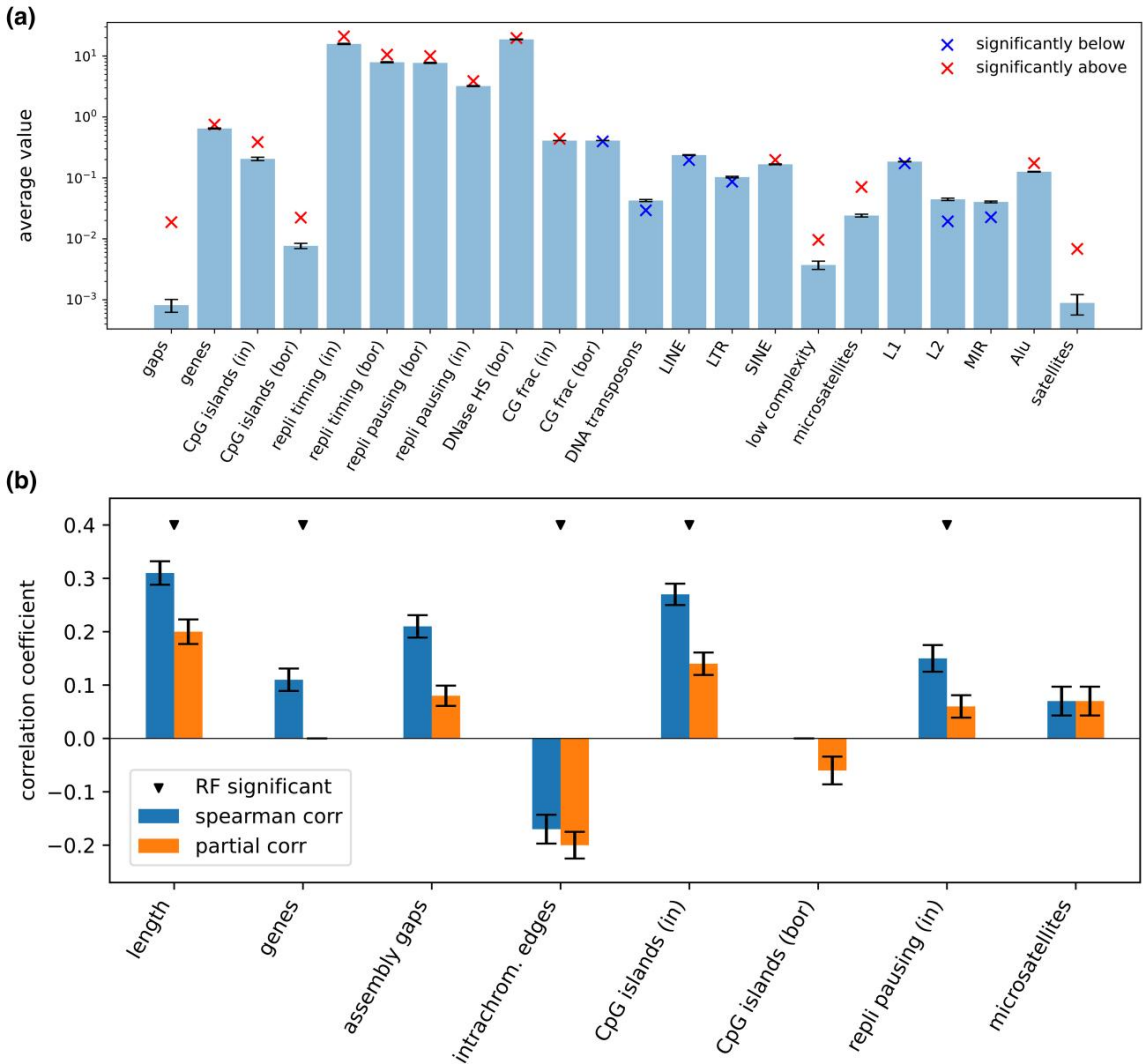
a) Characteristics of duplicated regions compared to the genome average. The barchart represents mean values (or frequencies where relevant) of various genomic features measured in randomly shuffled genomic intervals. The crosses are values that we observe for original duplicated regions. We report only those features for which the arithmetic mean is not belonging to the empirical background distribution of means measured in the shuffled samples (if Bonferroni adjusted *p*-value <0.001). For each feature, we used an arithmetic mean and sample variance measured on the shuffled samples as parameters of the background distribution. b) Characteristics of duplicated regions significantly associated with the number of duplication events inferred from the MST of primary duplications. Only features with either significant nonzero regular Spearman’s or partial correlation coefficient are reported (see Methods for details). The error bars show 95% confidence intervals. Triangles mark features which have statistically significant positive feature importance values in the random forest model measured with permutation tests (see Methods for details).

Firstly, we studied if various characteristics of duplicated regions differ from those observed at random genomic sites. We compared duplicated regions against the genome background distribution without taking into account the number of duplication events we predicted. Multiple characteristics of duplicated regions were either significantly larger or smaller than expected from the null hypothesis of random distribution (Fig. 3a). It is not surprising given the fact that duplications are distributed nonuniformly in the human genome. For example, we observed that assembly gaps are enriched at flanking regions (because large complex duplications are hard to assemble), duplicated regions are located in late replicating regions or/and in those where DNA polymerase slows down. Also, there was an enrichment of CpG islands and genes inside of duplicated regions. The same pattern was observed when we filtered out duplicated regions associated with assembly gaps (supplementary fig. S5, Supplementary Material online).

**Fig. 4.**
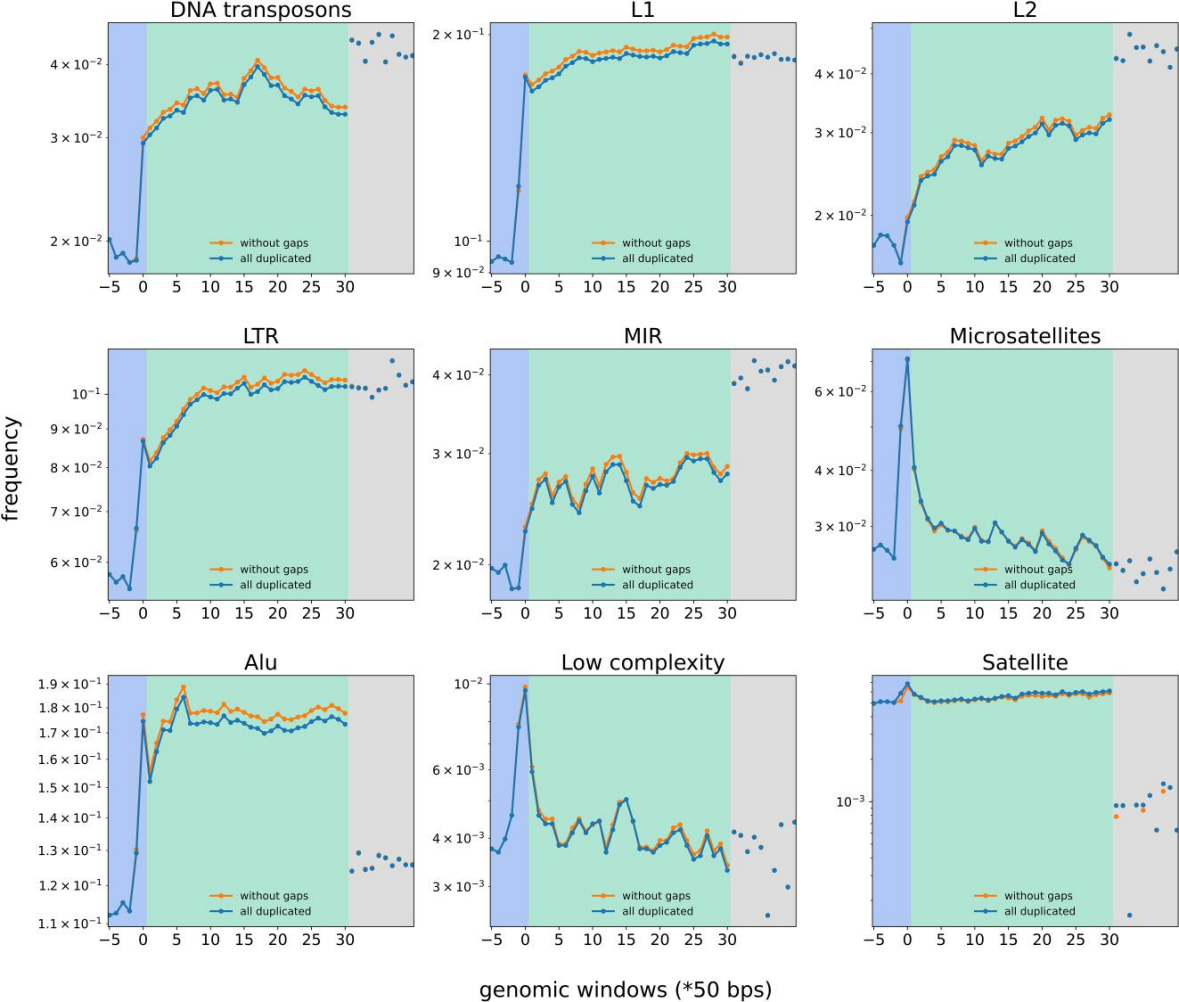
The distribution of different repeat families relative to the breakpoints of duplicated regions. Each point represents the frequency of a specific repeat family in two 50 bp genomic windows (downstream and upstream). Frequency is calculated as the number of repeats falling within the windows divided by the number of windows. Pairs of windows were shifted relative to the breakpoints in a nonoverlapping manner: inside of duplicated regions (5 measurements colored in blue), outside duplicated regions (30 measurements colored in green), and were placed at random genomic positions (10 measurements in the gray area). Two lines correspond to all duplicated regions (blue) and those without neighboring assembly gaps (orange). The first group of repeats (panels from DNA transposons to *MIR* repeats) includes those depleted inside of duplicated regions with no enrichment observed around the breakpoints. The second group (panels from satellites to low complexity repeats) includes repeats that are enriched at the very breakpoints of duplicated regions or in nearby windows outside (satellites, microsatellites, *Alu* and low complexity repeats).

We studied in detail how high-copy repeats are distributed relative to duplicated regions breakpoints (inside, outside, or at flanks of duplicated regions). To do this, we moved sliding nonoverlapping windows of length 50 bps inside and outside of breakpoints of duplicated regions and counted the number of overlapping repeats (see Methods for details). Also, for each repeat class, we did 10 more measurements in random genomic positions. As a result, the distributions were different for different repeat classes. These could be divided into two groups. Repeats in the first group include: DNA transposons, *LTRs*, *L1*, and *L2* repeats from the LINE family and *MIR* repeats from SINE. These are depleted inside of a duplicated region and their numbers grow when we move further outside from a duplicated region borders (Fig. 4). Repeats in the second group are enriched in a close proximity outside of duplicated regions, especially at the very breakpoints. This group includes: satellites, microsatellites, *Alu*, and low complexity repeats (illustrated at Fig. 4 subplots, starting from the satellites on). The low complexity repeats are composed of polypurine or polypyrimidine repeated stretches, or regions of high A/T or G/C content. The second group of repeats is likely responsible for genomic instability that leads to duplication events. It is unlikely that observed biases are attributed to a nonuniform SDs distribution in the genome (concentration of SDs in subtelomeric or subcentromeric regions, proximity to assembly gaps, etc.). Firstly, because repeats from the second group are depleted inside of duplicated regions and at a relatively small distance from them and, secondly, because exclusion of duplicated regions flanked by the assembly gaps did not change the observed distributions (orange line at Fig. 4).

#### Prediction of Genomic Features Associated with Increased Duplication Rates

We trained a random forest (RF) model to find genomic features associated with the number of duplications a region had undergone. We estimated the feature importance that is assigned to predictor variables by the RF algorithm with additional rounds of permutations (see Methods or Altmann et al. 2010). As a result, the following genomic features were important for the number of duplications prediction: length of a duplicated region (emp. *p*-value <0.001), fraction of intrachromosomal edges (emp. *p*-value <0.001), number of overlapping genes (emp. *p*-value =0.023), and CpG islands (emp. *p*-value =0.0025), replication pausing (emp. *p*-value =0.025) (the last two measured inside of a duplicated region). To clarify the type of dependence between each feature and the number of duplications, we calculated partial Spearman’s correlation coefficient controlling for all other features as possible confounding variables and the regular Spearman’s correlation (Fig. 3b). One can interpret these coefficients in the following way: a regular correlation coefficient represents the dependence we would observe without considering other correlated features. However, it could be a result of other confounding correlations that are taken into account when the partial correlation is calculated. It shows the effect of one variable on another independent of other variables.

The partial and regular Spearman’s correlation coefficients are significant and positive for the length of a duplicated region, number of CpG islands (inside), presence of assembly gaps in proximity and replication pausing (inside), while negative for intrachromosomal edges fraction. As we mentioned earlier, the loci with reduced DNA polymerase velocity could be associated with genomic duplications (Chen et al. 2015). Our results support this hypothesis: the association of replication pausing and duplication frequency is reproduced by several our approaches (Fig. 3b). On the other hand, we did not find evidence of associations with late or early replicating regions. Positive correlation of the number of overlapping genes also has biological sense since segmental duplications were responsible for gene families propagation and evolution in the human lineage. The increased G/C and CpG dinucleotide content has previously been observed in segmental duplications, but the association of CpG islands with actively duplicating regions has not been described yet. Among high-copy repeats only microsatellites were associated with increased duplication rates based on our correlation tests. No other associations with repeat families were observed.

### Analysis of Segmental Duplication Signatures

We considered a scenario where several mechanisms for the accumulation of SDs act in a genome. Each one results in SDs of different genomic characteristics. We applied nonnegative matrix factorization (NMF) to find signatures of duplication processes (see Methods). NMF is a method of approximate matrix factorization into product of two matrices with all nonnegative elements. These matrices are interpreted as signatures matrix (one with characteristic patterns of some process) and weights matrix (with impacts of each signature at each sample) (Paatero and Tapper 1994). This method allows to detect characteristic patterns of SDs accumulation if present, without any *a priori* knowledge about duplication mechanisms. We applied NMF to the matrix of duplicated regions characteristics in 10 Mbp genomic windows. For each genomic window, we took duplicated regions that overlap it, extracted associated genomic features (see supplementary table 2, Supplementary Material online) and summed them up. To predict the optimal number of signatures, we used several approaches listed in the Methods section. As a result, NMF with s=3 signatures was the most consistent for observed data from the human genome. We will further denote them as signatures s1–s3. We assigned one of our three signatures to each individual duplicated region as described in Methods section. As a result we got 550, 3,314, and 2,792 duplicated regions assigned to s1, s2, and s3, respectively.

**Fig. 5.**
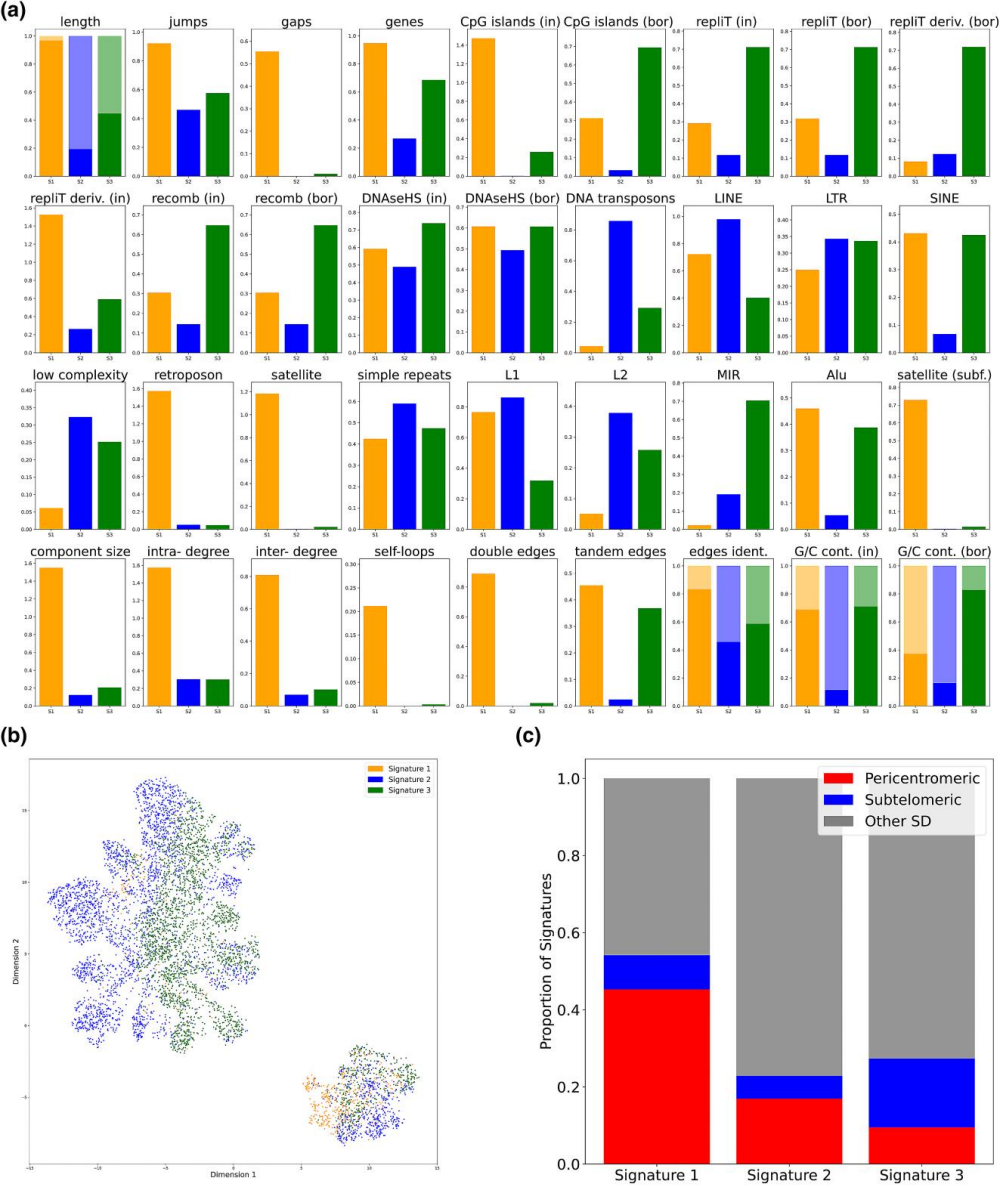
a) Characteristics of signatures s1, s2, and s3 colored in orange, blue, and green, respectively. Four features (length, sequence identity, G/C content inside, and at flanks) are binned to below (lighter) and above (darker color) their mean values. b) The UMAP embedding of duplicated regions features. The smaller cluster corresponds to the points belonging to the giant component, while the larger one includes all other connected components. Points belonging to the same signature tend to colocalize (signatures are indicated by the colors). In particular, s1 duplicated regions are enriched in the giant component cluster. c) The fraction of duplicated regions belonging to different signatures in different parts of chromosomes. Segmental duplications within 5 Mbp of a telomere or centromere are labeled as subtelomeric or pericentromeric, respectively.

The signature s1 corresponds to long duplications that overlap genes and CpG islands and are enriched in the giant component and in difficult-to-assemble regions of the genome (Fig. 5a). All graph attributes of actively duplicating regions are also associated with s1 (e.g. double edges, self-loops, and high node degrees). On average, s3 is shorter, but it also contributes to a significant number of gene duplications. These duplications are located in later and slower replicating genomic regions. All three signatures differ in the major repeats represented on their flanks. Signature s3 is dominated by SINE (especially *MIR* repeats), s1 by *Alu* repeats, nonautonomous retrotransposons and satellites, while s2 is associated with DNA transposons and LINE repeats (both *L1* and *L2*). The duplicated regions belonging to the different signatures are nonrandomly distributed with respect to telomeres and centromeres (Fig. 5c and supplementary fig. 8, Supplementary Material online). The s1 signature is enriched within pericentromeric regions, whereas the fraction of subtelomeric duplications is highest for s3. This explains the association of the s1 signature with satellite repeats, which are enriched in subtelomeric regions.

## Discussion

To study SDs in the human genome, we constructed the SD network. The nodes of it represent genomic regions involved in duplications and edges indicate the presence of an alignment between two corresponding regions. Earlier we observed that duplication rates grow with the number of copies of a specific duplicated region, which we called preferential duplication rates (Abdullaev et al. 2021). Here, we further examined the biological characteristics that could affect duplication rates.

To estimate the number of duplications that each node had undergone, we assigned edges weights inversely proportional to the number of shared neighbors and constructed the MST that goes through edges of minimal overall weight (putative primary duplications). The algorithm was validated on PCM synthetic networks and some additional indirect evidence was used to estimate its quality. The network-based approach allowed us to study segmental duplications as a whole thus revealing some universal biological principles of SDs evolution at the genome-wide scale.

We found that genomic characteristics are different in duplicated regions when comparing them to the rest of the genome. This can be explained by highly nonrandom distribution of SDs in the human genome. For example, assembly gaps are enriched at duplicated regions breakpoints. This stems from the fact that complex duplication events are harder to assemble than nonredundant sequences and therefore are often not fully “embedded” in a genome assembly. The gene content was higher in duplicated regions, consistent with an earlier report (Zhang et al. 2005). Moreover, it was positively correlated with the number of duplications a region had undergone. This may be because multiple gene duplications are responsible for innovations in the human genome evolution. In accordance with observations by Vollger et al. (2023) where increased CpG content was observed in SDs, we find an enrichment of CpG islands in duplicated regions. However, we additionally identified that the number of overlapping CpG islands is positively correlated with duplication rates. We would like to note that even though the distribution of CpG islands is correlated with both the G/C content and gene density, the partial correlation coefficients and the RF importance values have been used to predict a true associations not affected by confounding factors. We observed that duplicated regions are located in late and slower replicating parts of the genome in comparison with the genome average in agreement with Chen et al. (2015). However, we also found that duplication rates are higher in slower replicating regions. In other words, based on our analysis it appears that replication pausing not only causes duplication events, but also accelerates the duplication rate of already duplicated regions.

The composition of repeat families is quite different in genomic windows proximal to the breakpoints of duplicated regions. Those repeat families which are depleted inside of duplicated regions and around the breakpoints, while their fraction is per average higher in the rest of the genome include: DNA transposons, *L1*, *L2*, *LTR*, and *MIR* repeats. We suggest that these repeats are rarely involved in duplication events. Another group includes repeats that are enriched at breakpoints thus, likely, making genome susceptible to duplications. These are satellite, microsatellite, *Alu*, and low complexity repeats. It is unlikely that observed distributions relative to breakpoints are attributed to a nonuniform SDs distribution in the human genome (concentration of SDs in subtelomeric or subcentromeric regions, proximity to assembly gaps, etc.). Firstly, in most cases frequencies of repeats change dramatically when moving short distances away from breakpoints which is not expected if only a biased placement of SDs play a role. Also, repeats distributions did not change substantially when we excluded duplicated regions proximal to assembly gaps.

We further studied characteristic patterns (signatures) of the duplication process. We observed three signatures with various biological characteristics. Duplications belonging to signatures s1 and s3 are concentrated in pericentromeric and subtelomeric regions, respectively. s1 duplications are long and complex multicopy loci which are overrepresented in the giant component of the SD network. These SDs are often flanked with assembly gaps, satellite repeats, and nonautonomous retrotransposons. On the other hand, s2 duplications are much shorter, have much lower C/G content and are dominated by other groups of repeats at flanks: DNA transposons, *L1* and *L2*. The s3 duplications are distributed in interstitial manner in regions of higher recombination rates and slower replication timing with *SINE* repeats being enriched in their flanks. We suggest that these duplications likely originate from SINE-based nonallelic homologous recombination. These SDs are responsible for gene duplications and thus play an important evolutionary role.

Representing segmental duplications as a complex network opens up many research possibilities. For example, one can study the growth and evolution of a network for any well-annotated genome. We have shown that by analyzing the community structure of the SD network without any additional sources of information, one can in principle make predictions about the characteristic of actively duplicating loci (core duplicons) for a genome of interest with well-annotated SDs. Here, we used features of the SD network to predict, on a genome-wide scale, edges corresponding to real duplication events. Normally, reconstruction of a duplication history is a complicated task that is mostly done on a small scale: tracing the phylogeny of core duplicons or reconstructing complex events of a single locus, etc. The network representation allowed us to estimate the number of duplication events and thus find attributes associated with the duplication rate, which would otherwise be difficult to predict on a genome-wide scale.

## Methods

### Genomic Data

The list of annotated SDs for the GRCh38 reference genome and all genomic features we considered in association tests were downloaded from the UCSC genome browser website (https://genome.ucsc.edu). For our analysis, we disregard segmental duplications belonging to the sex chromosomes.

The genomic features we used include: replication timing, recombination rates, openness of chromatin, genome assembly gaps, CTCF sites, coordinates of a duplicated region, G/C nucleotides content, number of gene overlaps, repeat overlaps, and CpG island overlaps (see supplementary table S2, Supplementary Material online). UCSC LiftOver tool was used to transfer coordinates from *hg19* to *hg38* where needed (Kent et al. 2002). The feature values were measured either inside of a duplicated region (between its breakpoints) or in flanking regions of length 50 bps padding the duplicated regions on both sides (“Position” column at supplementary table S2, Supplementary Material online). Some of the features are cell line specific, however, the cell lines where segmental duplications happened belong to the germline. In absence of a relevant data from the germline, we picked the source of the data as listed in the “Cell line” column. The feature types include: counts (repeats, assembly gaps, etc.), mean values in corresponding genomic intervals (replication timing, recombination rate, etc.) and fractions (fraction of G/C nucleotides, intrachromosomal edges from all neighbors of a node). Other than that, a span of replication timing, i.e. the difference between maximal and minimal timing values in a genomic interval, was measured and used as a proxy for the replication pausing. For those features where flanking regions are studied, we did not distinguish between flanks and used the sum (or mean) of two values.

High-copy repeats distribution relative to duplicated regions breakpoints was studied by counting their numbers in sliding nonoverlapping windows. These windows were first placed proximal to breakpoints of a duplicated region (coordinates: [0;49] where 0 corresponds to a breakpoint position). Then moved 30 steps away ([0;49], [50;99], …, [1,450;1,499]) and five steps inside of a duplicated region ([−250;−201], [−200;−151], …, [−50;−1]). These sliding windows moved symmetrically (as a pair) relative to breakpoints on both sides of a duplicated region and counted the number of repeat overlaps in each pair of windows. Moreover, to study the background distribution the sliding windows coordinates were randomly shuffled throughout the human genome 10 more times.

### Network Analysis

We generated a network of SDs where each node represents a duplicated region while edges connect nodes if an alignment between two corresponding duplicated regions exists. We used this network after trimming multiple edges between any pair of nodes and self-loop edges (edges from a node to itself). To each edge of the SD network, we assigned a weight as described in the “Results” section. Those weights are reversely proportional to the fraction of neighbors that two nodes share. We run the Kruskal’s algorithm to reconstruct the MST on the weighted SD network (Kruskal 1956).

To evaluate the accuracy of our approach, we generated PCM synthetic networks. We simulated the PCM network growth and kept information on edges status (“primary” edges representing duplication events or “secondary” ones). We considered several centrality measures assigned to nodes of the SD network (Table 1) and estimated an accuracy of each of them when applied to our task.

To test our duplication reconstruction method on more realistic simulated networks, we added two processes to the original PCM model. First, each edge has a sequence identity level that is equal to 1.0 at the time of duplication and gradually decreases over time. When an identity is below 0.9, the edge is removed from the network. Edges inherited by a “daughter” node also inherit identity levels from a “mother” node. We run simulations with the per-base mutation rate ($r_{m}$) comparable to the per-base duplication rate ($r_{d}$) in agreement with the literature (Massip and Arndt 2013; Dennis and Eichler 2016). We operated in the interval $\frac{r_{d}}{r_{m}}\in [1.0;\;2.0]$. In other words, when $\frac{r_{d}}{r_{m}}=1.0$, we expect about 5% of basepairs being mutated. Both papers considered duplications that are fixed in the human population, so this estimate is more relevant to the growth of the SD network than the rate of *de novo* CNVs. Moreover, it is also the highest mutation rate estimate and therefore the most perturbing to the simulated network.

Second, we added a process that represents a nonallelic gene conversion (NAGC) in our simulation. At each time step, any edge can change it’s identity back to 1.0 with probability $r_{\mathrm{GC}}$. Since gene conversion results in the copying of the DNA sequence from a donor locus to an acceptor locus we also included this process in our simulation. When a NAGC event happens, sequence identity levels of all shared neighbors are inherited from the donor to the acceptor node. Normally gene conversion takes place between homologous sequences with a relatively high degree of sequence identity: the vast majority of described NAGC events took place between sequences with >90% identity (Vollger et al. 2023). So in our simulations edges do not appear between nodes that did not have edges before or when an edge has already been lost. In other words, if the sequence identity of an edge falls below 0.9, we consider that this edge evades potential gene conversion events. However, an alternative scenario without evasion was also tested with the same resulting performance of the MST algorithm. We report results of simulations where the NAGC rate $r_{\mathrm{GC}}$ is set such that 34% of all edges are affected by it at the end of the simulation, in agreement with the estimates from Vollger et al. (2023). However, the performance of our MST prediction algorithm was stable even when much higher $r_{\mathrm{GC}}$ values were tried.

We applied the permutation tests when studied whether edges of interest (high sequence identity ones or ones with matching breakpoints) are enriched in the MST. By doing 10,000 sampling rounds from all edges of the SD network, we generated a background distributions to compare with the real values.

### Core Duplicons Analysis

The mosaic duplications associated with the core duplicons were downloaded from Jiang et al. (2007). The UCSC LiftOver tool was used to transfer coordinates from *hg17* to *hg38* (Kent et al. 2002). Mosaic duplications that overlapped more than one node of the SD network were assigned to the node with the longest overlap by sequence. We used the label propagation algorithm described by Raghavan et al. (2007) and implemented in the Julia Graphs package to predict the communities of nodes in the SD network.

### Associations Analysis

To predict genomic features associated with the number of duplications we used several machine-learning algorithms. The applied algorithms include: linear regression, support vector regression (SVR), decision trees, and RF. All of them reached a similar quality of predictions with the maximal % of variance explained $R^{2}=30.5\%$ observed for the RF (estimated based on a 5-fold cross-validation). To estimate a statistical significance of RF feature importance values, we used a special permutation test described by Altmann et al. (2010). Multiple rounds of permutation of a response variable with consequent runs of random forest algorithm allow to approximate a null distribution of importance values for each feature when no interaction between a predictor and response variables exist. The empirical *p*-value of a feature importance is equal then to the fraction of all permutations where the corresponding feature obtained higher importance value. The resulting empirical *p*-values allow overcoming biases described in Altmann et al. (2010).

We calculated partial Spearman’s correlation coefficient between each feature and the number of duplications controlling for all other features as possible confounding variables. We used pingouin python package to measure partial correlations. Pingouin uses the method described in Vallat (2018) and Kim (2015) to calculate the semi-partial correlation coefficients and associated *p*-values. The confidence intervals for the correlation coefficients are estimated using the Fisher transformation.

### Nonnegative Matrix Factorization

Nonnegative matrix factorization (NMF) is a matrix decomposition method. NMF decomposes an n×f matrix named $A_{\left(n,f\right)}$ into a product of two matrices, namely $W_{\left(n,k\right)}$ and $H_{\left(k,f\right)}$. The NMF is computed using a multiplicative iterative algorithm. The Kullback–Leibler divergence function was used as the loss function.

We normalized our data by scaling the columns between 0 and 1 (the min–max scaler), so that none of the features dominate the NMF signatures due to their magnitude. Some features were binned relative to the mean before being summed. These include: length, G/C content (inside and at flanks), and sequence identity. For each nonoverlapping 10 Mbp genomic window, we calculated the sum of all genomic features. We then applied NMF to the matrix, where each row corresponds to a 10 Mbp window and the columns are genomic features. After predicting the signatures, we used linear regression with nonnegative coefficients to assign each duplicated region to one of the three signatures. The one with the highest weight was considered to be responsible for the formation of a duplicated region of interest. For supplementary fig. 6, Supplementary Material online, we classified our duplicated regions as subtelomeric and pericentromeric if they were located at a distance of <5 Mbp from the telomere or centromere, respectively.

The uniform manifold approximation and projection (UMAP) embedding hyperparameters were set to 100 n_neighbors and the minimum distance as 1 (McInnes et al. 2018).

#### Finding the Number of Signatures *k*

To determine the optimal number of signatures *k*, we experimented with synthetic datasets where *k* is known a priori. We used two main methods: Fogel and Young’s volume-based method (FYV) and the signature stability approach of Fogel et al. (2007).

The FYV method, a volume-based approach, measures the determinant of the signature matrix to estimate the diversity of the signatures. A higher determinant value typically indicates a better distribution of signatures and helps to identify the number of different patterns present in the data. In our analysis, FYV suggested an optimal *k* of 4.

The signature stability method assesses how consistent the extracted signatures are across multiple runs with random initialization of the signature and weight matrices. Stability is assessed by comparing the signatures from each run: highly similar signatures across runs indicate that the number of signatures is well suited to the underlying structure of the data. This method indicated k=2 as the optimal choice.

After comparing these automated predictions with manual evaluation of the generated signatures, we chose k=3 as the best compromise between discriminability and stability. A summary of the methods is given in supplementary fig. S6, Supplementary Material online.

## Supplementary Material

evaf011_Supplementary_Data

## Data Availability

The scripts utilized in our analysis are available in the GitHub in the repository: https://github.com/abdeldar/SD_network. There we also give instructions to download and install the Julia programming language and all used packages. One can use the script to build the network of segmental duplications for any well-annotated genome. All genome annotations were downloaded from open access databases (see Methods section).
